# Effect of bilirubin on cytochrome *c *oxidase activity of mitochondria from mouse brain and liver

**DOI:** 10.1186/1756-0500-3-162

**Published:** 2010-06-09

**Authors:** Safarina G Malik, K Astrid Irwanto, J Donald Ostrow, Claudio Tiribelli

**Affiliations:** 1 Eijkman Institute for Molecular Biology, Jl. Diponegoro 69, Jakarta 10430, Indonesia; 2 GI/Hepatology Division, Dept. Medicine, University of Washington, Seattle, WA, 98195, USA; 3 CSF AREA Science Park and Dept. ACADEM, University of Trieste, 34012, Trieste, Italy

## Abstract

**Background:**

The unbound, free concentration (B_f_) of unconjugated bilirubin (UCB), and not the total UCB level, has been shown to correlate with bilirubin cytotoxicity, but the key molecular mechanisms accounting for the toxic effects of UCB are largely unknown.

**Findings:**

Mouse liver mitochondria increase unbound UCB oxidation, consequently increasing the apparent rate constant for unbound UCB oxidation by HRP (Kp), higher than in control and mouse brain mitochondria, emphasizing the importance of determining Kp in complete systems containing the organelles being studied. The *in vitro *effects of UCB on cytochrome *c *oxidase activity in mitochondria isolated from mouse brain and liver were studied at B_f _ranging from 22 to 150 nM. The results show that UCB at B_f _up to 60 nM did not alter mitochondrial cytochrome *c *oxidase activity, while the higher concentrations significantly inhibited the enzyme activity by 20% in both liver and brain mitochondria.

**Conclusions:**

We conclude that it is essential to include the organelles being studied in the medium used in measuring both Kp and B_f_. A moderately elevated, pathophysiologically-relevant B_f _impaired the cytochrome *c *oxidase activity modestly in mitochondria from mouse brain and liver.

## Backgrounds

Unconjugated bilirubin (UCB) at low concentrations is a potent antioxidant [1,2] that is neuroprotective [3], while higher levels of UCB may damage neurons and astrocytes [3-6], resulting in bilirubin-induced neurological dysfunction (BIND) in some neonates with severe unconjugated hyperbilirubinemia. It has been shown that the unbound, free concentration (B_f_) of unconjugated bilirubin (UCB), and not the total UCB level, correlates with bilirubin cytotoxicity [7], but the key molecular mechanisms accounting for the toxic effects of UCB are largely unknown. Although the primary subcellular targets for the toxic effects of UCB are not fully identified, mitochondria are believed to be particularly vulnerable [4,8-12]. However, with few exceptions [10,13], most prior research was performed at unbound UCB concentrations (B_f_) orders of magnitude higher than its hypothesized upper normal limit of 20 nM (1.2 μg/dL) in plasma of term infants [14]. In addition, most studies used unpurified commercial bilirubin, rendering uncertain whether the observed toxic effects were due to UCB itself, or to contaminants (especially lipids).

Here we demonstrate the need to include the organelles being studied in the medium used in measuring both Kp and B_f_. This study further describe the *in vitro *effect of UCB, at B_f _ranging from 22 to 150 nM, on cytochrome *c *oxidase (E.C. 1.9.3.1) activity in mitochondria isolated from mouse liver and brain. Cytochrome *c *oxidase is a crucial enzyme in aerobic energy metabolism, serving as the final electron acceptor complex in the mitochondrial electron transport. It catalyzes electron transfer from cytochrome *c *to molecular oxygen, reducing the latter to water, and yields substantial energy that drives the formation of a proton gradient that is then employed to synthesize cellular ATP [15]. The absence, deficiency or malfunction of this enzyme in human leads to a number of serious disease states [16]. The results obtained in this study are relevant to the *in vivo *impairment of mitochondrial function by elevated plasma levels of UCB.

## Materials and methods

### Materials

Bilirubin (Sigma Chemical Co.-Aldrich, Milan, Italy) was purified using the method of McDonagh and Assisi [17]. Phenylmethanesulfonyl Fluoride (PMSF), p-Aminobenzamidine dihydrochloride (PAB HCl), ε-Amino-n-Caproic Acid (ε-ACA), n-dodecyl β-D-maltoside, L-ascorbic acid, horseradish peroxidase (Type 1 HRP, EC.1.11.1.7), cytochrome *c *from horse heart, and Bovine Serum Albumin (BSA) fraction V (Fatty-acid free) were purchased from Sigma Aldrich (St. Louis, MO, USA). Reagent grade hydrogen peroxide (H_2_O_2_, 30% wt/vol), hydrochloric acid (HCl, 37%), chloroform, sodium hydroxide and sucrose were purchased from Merck (Darmstadt, Germany). EDTA (disodium salt, dehydrate, crystal) was purchased from BDH (Dorset, England). Tris was purchased from Invitrogen (Carlsbad, CA, USA). HEPES was purchased from Gibco BRL (Grand Island, NY, USA).

### Reagents

Purified UCB was dissolved in chloroform (0.5 mg/mL) and the concentrations (μg/mL) determined spectrophotometrically as A_453 nm _× 0.974 × dilution factor [17]. UCB was divided into 50 μg and 300 μg aliquots, and the chloroform evaporated under nitrogen at <65°C, and stored at -20°C until used. UCB was dissolved either in 0.01 N NaOH for Kp measurement or 0.5% vol/vol DMSO for B_f _measurement and Complex IV Assay. H_2_O_2 _(1%) was prepared freshly each day by diluting 33.3 μL of 30% H_2_O_2 _with 967 μL of potassium phosphate buffer, pH 7.4. HRP stock solution (1 mg/mL) was prepared by dissolving 1 mg HRP in 1 mL potassium phosphate buffer, pH 7.4, and stored at -20°C. Mitochondria isolation buffers are as described in [18] for liver mitochondria and in [19] for brain mitochondria. Cytochrome *c *was reduced by L-ascorbate according to the method of [20]. Full reduction of cytochrome *c *was confirmed by measuring the absorbance at 550 and 560 nm.

### Methods

Isolation of mitochondria from mouse liver (LM) and brain (BM) were obtained by sacrificing six BALB/c mice (8-10 weeks) by decapitation (approved by the Eijkman Institute Research Ethics Commission). LM isolation was as in [18], and brain mitochondria (BM) isolation was as in [19]. Mitochondrial protein concentration was determined by the modified Lowry Protein Assay [21]. Determination of unbound bilirubin (B_f_) was performed using the minimally-diluted peroxidase method [22] with modification [23]. Unbound bilirubin was oxidized by peroxide (H_2_O_2_) in the presence of horseradish peroxidase (HRP) with first-order kinetics, while albumin-bound bilirubin was not [24]. The preliminary standardization of the rate constant (Kp) for oxidation of unbound UCB was done by measuring the decrease in A_440 _following addition of HRP and H_2_O_2 _in a system containing buffer, UCB, and 100 μg of brain or liver mitochondria in the absence of albumin [22-24]. Unbound bilirubin (B_f_) was determined in a complete system containing buffer, BSA, UCB, and 100 μg of brain or liver mitochondria, by measuring the decrease in A_468 _in the presence of HRP and H_2_O_2_. B_f _was calculated from Kp, the HRP concentration, and the initial change of light absorbance peak at 468 nm over time [23,24]. Triplicate determinations were performed for each measurement, and Kp or Bf was determined from the average of at least three mitochondrial preparations.

Cytochrome *c *oxidase activity was measured at 37°C as described previously, using 100 μg of brain or liver mitochondria [20,25], and expressed as percent activity normalized to control incubations at the same BSA concentration but without UCB. The oxidation of cytochrome *c *by cytochrome *c *oxidase was followed spectrophotometrically at 550 nm for 30 s, assuming ε^1 cm ^= 19,600 per mol for horse heart cytochrome *c *[26]. The effect of purified UCB on the activity of cytochrome *c *oxidase was studied at low (22-31 nM and 60-61 nM) and high (92-107 nM and 142-150 nM) B_f _concentrations.

Results for LM and BM were the average of triplicate determinations performed at each B_f _level. All data are expressed as mean ± SD of at least 3 experiments obtained in 3 different preparations of mitochondria. Statistical differences were calculated according to the two-tailed Student's t-test, performed on the basis of equal or unequal variance as appropriate. *p *values lower than 0.05 were considered statistically significant.

## Results and Discussion

### Liver mitochondria increase the oxidation of unbound UCB

Measurement of the rate constant for oxidation of unbound UCB by HRP (Kp, 15-20 repeats) was performed both in the absence and presence of BM or LM; controls were the assay mixture without mitochondria. Kp determination was needed to calculate unbound bilirubin concentration [23,24]. The mean control Kp values were unaffected by addition of BM, but were 1.7 times higher in the presence of LM (Table 1). The B_f _calculation was, therefore, based on the Kp value obtained with each experimental condition (control, with LM, and with BM).

**Table 1:** Effect of brain mitochondria (BM) and liver mitochondria (LM) on rate of peroxidation of unbound UCB by HRP (Kp)

	Kp	
	(mL·μg^-1^·min^-1^)	*vs *control (*p*)
**Control **^*a *^(n = 22)	0.406 ± 0.01	
**BM **^*b *^(n = 15)	0.399 ± 0.02	0.183
**LM **^*c *^(n = 16)	**0.699 ± 0.03**	< 0.001

Kp was determined by method described in [23], to measure the coupled constant rate of bilirubin oxidation by HRP. ^*a *^**Control**, no mitochondria; ^*b *^**BM**, mouse brain mitochondria; ^*c *^**LM**, mouse liver mitochondria.

As reported in Table 2, addition of 0.5 mM potassium cyanide (KCN), a cytochrome *c *oxidase inhibitor, decreased Kp to control values (p < 0.01) in the presence of LM; the decrease was even greater when the KCN concentration was increased to 1.0 mM. The dose-dependent Kp inhibition induced by KCN was much less prominent in assay mixtures containing BM.

**Table 2:** KCN inhibition of rate of peroxidation of unbound UCB by HRP (Kp)

**Relative Kp**^***a***^		
	***vs *control**	***vs *LM^***b ***^or BM^***c***^**

**Control **(n = 18)	100 ± 3.1	
0.5 mM KCN (n = 12)	84.7 ± 5.8 (*p *< 0.001)	
1.0 mM KCN (n = 11)	73.4 ± 5.8 (*p *< 0.001)	

**BM**^***b ***^(n = 11)	99.2 ± 4.4 (*p *= 0.618)	
0.5 mM KCN (n = 6)	88.7 ± 3.3 (*p *< 0.001)	89.4 ± 3.4 (*p *< 0.001)
1.0 mM KCN (n = 6)	72.1 ± 6.8 (*p *< 0.001)	72.1 ± 6.9 (*p *< 0.001)

**LM**^***c ***^(n = 12)	172 ± 6.2 (*p *< 0.001)	
0.5 mM KCN (n = 6)	102 ± 8.1 (*p *= 0.580)	59.4 ± 4.7 (*p *< 0.001)
1.0 mM KCN (n = 6)	83.8 ± 9.3 (*p *< 0.001)	48.8 ± 5.4 (*p *< 0.001)

^*a *^Percent of Kp values normalized to control (no mitochondria); ^*b *^**BM**, mouse brain mitochondria; ^*c *^**LM**, mouse liver mitochondria.

The higher Kp values and the decrease of Kp due to KCN addition in the presence of LM indicate that LM contain higher levels of intrinsic peroxidases than BM, confirming previous studies [27]. It has been reported that BM contains bilirubin oxidase, which also has been detected in other organs including liver [28,29]. Other peroxidases known to be present in mitochondria include phospholipid hydroperoxide glutathione peroxidase (PHGPx) [30], glutathione peroxidase (GPx), catalase (CAT) [30,31], and peroxiredoxin (Prx) III [32], which like other hemoproteins, are inhibitable by KCN [29,31]. In LM, these intrinsic mitochondrial peroxidases contribute to the UCB oxidation measured in the presence of the added HRP, accounting for the almost 2× higher apparent Kp in the presence of LM compared to BM. These findings emphasize the importance of determining Kp in complete systems containing the organelles being studied.

### *Effects of B*_*f *_*on mitochondrial cytochrome *c *oxidase activity*

B_f _was varied by using various B/A molar ratios (r) and two different BSA concentrations (Table 3). As expected, the B_f _increased with r, with a tendency to slightly higher measured B_f _in systems containing LM compared to BM. Except for r = 0.35 (*p *= 0.215), these differences were significant (*p *< 0.005 for r = 0.2; *p *= < 0.005 for r = 0.5; *p *< 0.005 for r = 0.75).

**Table 3:** Measured unbound bilirubin (B_f_) concentrations at various ratios of [UCB]/[BSA]

	B_f _(nM)				
			
UCB/BSA Ratio, [BSA] μM	**Control**^***a***^	**BM**^***b***^	**LM**^***c***^	*p*-value (BM *vs *LM)	*p*-value (control *vs *LM)
**0.20, 60**	22.3 ± 3.4	22.6 ± 2.2	30.8 ± 2.2	*p *< 0.005	*p *< 0.005
**0.35, 60**	61.4 ± 2.3	59.6 ± 2.0	61.5 ± 4.3	*p *= 0.215, NS	*p *= 0.788, NS
**0.50, 60**	90.2 ± 2.6	93.4 ± 5.6	104 ± 4.6	*p *< 0.005	*p *< 0.005
**0.75, 30**	141 ± 3.9	142 ± 3.4	150 ± 6.3	*p *< 0.005	*p *< 0.005

B_f _concentrations were determined by the peroxidase method in potassium phosphate buffer (50 mM KH_2_PO_4 _- 50 mM K_2_HPO) containing 50 mM KCl, pH 7.4 [22,23]. ^*a *^**Control**, no mitochondria, ^*b *^**BM**, mouse brain mitochondria; ^*c *^**LM**, mouse liver mitochondria. Data are expressed as mean ± SD of at least 9 experiments.

The mean value of B_f _from 4-5 replicate tubes was utilized in assessing the effects of increasing B_f _on cytochrome *c *oxidase activity. As shown in Table 4, the exposure of mitochondria to B_f _of 22-31 nM and 60-61 nM was not associated with any significant effect on cytochrome *c *oxidase activity of either BM or LM as compared to control. In contrast, B_f _of 92-107 nM and 142-150 nM caused significant, comparable reductions (18-20%) of cytochrome *c *oxidase activity in both BM and LM; no significant difference was found between the inhibition observed at mean B_f _values of 100 and 146 nM. *In vivo *the brain and liver mitochondria are exposed to cytosolic fluid in which B_f _is much lower than in plasma [33]. These results suggest that cytochrome *c *oxidase activity is probably only minimally impaired at plasma unbound bilirubin concentrations found during neonatal jaundice. Given that cytochrome *c *oxidase is a crucial enzyme in aerobic energy metabolism, and deficiency or malfunction of this enzyme in human leads to a number of serious disease states, changes caused by high B_f _concentration may cause noticeable damage and alter the whole mitochondrial function. Others have reported recently that the cytochrome *c *oxidase activity in rat cortical neurons was decreased by approximately 50% due to exposure to 50 μM UCB in the presence of 100 μM HSA [34]. This gives an estimated B_f _of 119 nM [35], midway between the two highest B_f _values we studied. The difference in the degree of inhibition they observed might be due to the differences between rat and mouse mitochondria, as well as the differences of the systems used. Vaz *et al *[34] exposed cultured neuronal cells to UCB, then isolate the mitochondria afterwards, while this study directly exposed isolated mitochondria from whole brain consisiting of a mixture of cells, including neurons and glial cells. It has been reported that neurons respond differently from glial cells to bilirubin induced toxicity [29,36,37]. In particular, the rate of bilirubin oxidation was reported to be significantly lower in mitochondrial membranes from a pure neuronal source than membranes from either a glial source or a mixed neuronal/glial source [29], hence the higher sensitivity of neurons to UCB induced toxicity as compared to glial cells.

**Table 4:** Cytochrome *c *oxidase activity of mouse liver and brain mitochondria exposed to different free bilirubin (B_f_) concentrations

		**Percent Activity (%) *vs *control**^***a***^	
		
[UCB] **[HSA]**^***b***^	Range of B_f _(nM)	BM^*c*^	LM^*d*^
**0.20**	22-31	105 ± 3.1	101 ± 1.8
**0.35**	60-61	104 ± 8.9	99.1 ± 3.1
**0.50**	92-107	82.0 ± 4.5^*e*^	84.9 ± 3.5^*e*^
**0.75**	142-150	82.6 ± 1.0^*e*^	80.7 ± 5.8^*e*^

^*a *^Results are means of three repeats of at least three separate measurements of the activity of cytochrome *c *oxidase activity as percent of control (no UCB). ^*b *^Molar ratios of bilirubin to albumin (r). ^*c *^Mouse liver mitochondria. ^*d *^Mouse brain mitochondria. ^*e *^All *p *< 0.001 vs. B_f _= 22-31 nM, and *p *< 0.05 vs B_f _= 60-61 nM, respectively. No significant differences between: B_f _= 22-31 nM vs. B_f _= 60-61 nM (*p *= 0.936 for BM and *p *= 0.302 for LM); B_f _= 92-107 nM vs. B_f _= 142-150 nM (*p *= 0.791 for BM and *p *= 0.342 for LM); or BM vs. LM at any range of B_f _(*p *= 0.086, *p *= 0.372, *p *= 0.298, *p *= 0.605, respectively).

## Conclusions

We showed that, in the application of the peroxidase method, it is important to determine Kp, the first order rate constant for HRP-catalyzed UCB oxidation by peroxide in the absence of albumin; Kp is then used to calculate B_f_, in a complete system containing the organelles being studied. As demonstrated in this study, liver mitochondria increase the oxidation of unbound UCB, thus increasing the apparent Kp. As stated at the beginning of this paper, B_f _has been shown to correlate with bilirubin cytotoxicity. B_f _measurement using the peroxidase method could improve the clinical management of neonatal jaundice by better recognizing babies that need treatment and minimizing unnecessary intervention [38].

The present study, using purified UCB at pathophysiologically relevant B_f _concentrations, demonstrated that at modestly elevated B_f _concentrations (about 90 and 150 nM), UCB inhibits cytochrome *c *oxidase activity by about 20% in both brain and liver mitochondria from mice. Exposure to lower, but still elevated B_f _up to about approx. 60 nM did not significantly affect mitochondrial cytochrome *c *oxidase activity in either BM or LM. The lack of a progressive dose-response effect as B_f _increased from approx. 100 to 150 nM, as well as the modest 20% impairment in cytochrome *c *oxidase activity, suggests that impairment of mitochondrial oxidative activity might not be a major factor in the toxic effects of UCB on these organelles. The results do not negate the reports by others that UCB alters mitochondrial function in other ways [9,11], for example by altering mitochondrial membrane potential and triggering apoptosis by release of cytochrome *c *into the cytosol.

Our studies, done at B_f _levels compatible with those in the plasma of jaundiced neonates, did not confirm the suggested greater sensitivity of BM than LM to toxic effects of UCB, derived from studies at vastly higher UCB concentrations [7], although it is important to bear in mind that the source of the brain mitochondria used in this study are a mixture of various type of cells, not only the ones that are sensitive to bilirubin toxicity, such as neuronal cells, but also the less sensitive ones, such as glial cells [29]. These less sensitive cells might be masking the effect of bilirubin toxicity to the level similar to that of liver mitochondria.

The present work thus demonstrates the importance of comparing effects of UCB at comparable B_f _levels, measured using the peroxidase method. It also demonstrates the need to include the organelles in the medium used in measuring both Kp and B_f_.

## Competing interests

The authors declare that they have no competing interests.

## Authors' contributions

SGM designed the study, established and performed Bf determinations and cytochrome *c *oxidase assay, performed data analysis, wrote and revised the final manuscript. KAI performed Bf determinations, cytochrome *c *oxidase assay, data analysis, and wrote the manuscript. JDO and CT provided direction and oversight of the experiments, and helped revise the final manuscript. All authors read and approved the final manuscript.
